# Identification of major QTLs for yield-related traits with improved genetic map in wheat

**DOI:** 10.3389/fpls.2023.1138696

**Published:** 2023-03-17

**Authors:** Feifei Ma, Yunfeng Xu, Ruifang Wang, Yiping Tong, Aimin Zhang, Dongcheng Liu, Diaoguo An

**Affiliations:** ^1^ Center for Agricultural Resources Research, Institute of Genetics and Developmental Biology, Chinese Academy of Sciences, Shijiazhuang, China; ^2^ State Key Laboratory of North China Crop Improvement and Regulation, College of Agronomy, Hebei Agricultural University, Baoding, Hebei, China; ^3^ State Key Laboratory of Plant Cell and Chromosome Engineering, National Center for Plant Gene Research, Institute of Genetics and Developmental Biology, Chinese Academy of Sciences, Beijing, China

**Keywords:** wheat, high-density linkage map, QTL mapping, yield-related traits, the wheat 660K SNP array

## Abstract

**Introduction:**

Identification of stable major quantitative trait loci (QTLs) for yield-related traits is important for yield potential improvement in wheat breeding.

**Methods:**

In the present study, we genotyped a recombinant inbred line (RIL) population using the Wheat 660K SNP array and constructed a high-density genetic map. The genetic map showed high collinearity with the wheat genome assembly. Fourteen yield-related traits were evaluated in six environments for QTL analysis.

**Results and Discussion:**

A total of 12 environmentally stable QTLs were identified in at least three environments, explaining up to 34.7% of the phenotypic variation. Of these, *QTkw-1B.2* for thousand kernel weight (TKW), *QPh-2D.1* (*QSl-2D.2/QScn-2D.1*) for plant height (PH), spike length (SL) and spikelet compactness (SCN), *QPh-4B.1* for PH, and *QTss-7A.3* for total spikelet number per spike (TSS) were detected in at least five environments. A set of Kompetitive Allele Specific PCR (KASP) markers were converted based on the above QTLs and used to genotype a diversity panel comprising of 190 wheat accessions across four growing seasons. *QPh-2D.1* (*QSl-2D.2/QScn-2D.1*), *QPh-4B.1* and *QTss-7A.3* were successfully validated. Compared with previous studies, *QTkw-1B.2* and *QPh-4B.1* should be novel QTLs. These results provided a solid foundation for further positional cloning and marker-assisted selection of the targeted QTLs in wheat breeding programs.

## Introduction

Common wheat (*Triticum aestivum* L. 2n = 6x = 42, AABBDD) is one of the important grain crops in the world. Given the food security challenges caused by gradual decrease in arable land and rapid increase in global population, increasing the yield potential of wheat through high-yield breeding programs has been a major focus of most wheat breeders around the world. Considering the complex inheritance and significant influence of environment, yield improvement remains to be a huge challenge. Achievement of this goal will require full identification of promising yield-related loci in wheat.

Wheat yield comprises three main components, viz. spike number per plant (SNPP), kernel number per spike (KNS) and thousand kernel weight (TKW). Of these, SNPP and KNS are more easily influenced by environment (Li et al., 2018). Lots of quantitative trait loci (QTLs) for these two traits have been detected. Two genes *TaTEF-7A* and *GNI1* related to KNS and one gene *TaD27-7B* related to SNPP were cloned (Zheng et al., 2014; Sakuma et al., 2019; Zhao et al., 2020). For TKW, due to its higher heritability, numerous QTLs have been mapped on all 21 wheat chromosomes, and more than 20 genes related to kernel weight have been cloned (Jiang et al., 2011; Zhang et al., 2012; Guo et al., 2013; Chang et al., 2014; Dong et al., 2014; Zhang et al., 2014; Hanif et al., 2015; Jiang et al., 2015; Ma et al., 2016; Zhang et al., 2017b; Zhang et al., 2018b; Yan et al., 2019; Yang et al., 2019). KNS and TKW can be affected by spike length (SL) and kernel weight per spike (KWS). The gene *Q* played an important role in domestication of polyploid wheat because it pleiotropically influenced many important domestication-related traits, including rachis fragility, threshability, glume tenacity, spike architecture, plant height and flowering time (Simons et al., 2006). The gene *Q* was reported to control SL (Jiang et al., 2019), whereas genetic studies on KWS were not given enough attention previously. KNS can be further divided into kernel number per spikelet and total spikelet number per spike (TSS), which was composed of fertile spikelet number per spike (FSS) and sterile spikelet number per spike (SSS). A wheat ortholog of rice gene *APO1* was reported to be the best candidate gene for a locus on 7AL affecting TSS (Kuzay et al., 2019; Kuzay et al., 2022). Plant height (PH) was significantly related to SL and affected the harvest index (HI) and grain yield. More than 20 major genes of PH have been identified and designated as reduced height (*Rht*) genes (Peng et al., 2011; Chen et al., 2014; Lu et al., 2015; Zhang et al., 2017a; Ford et al., 2018; Chai et al., 2022; Xiong et al., 2022). PH and SNPP can affect biomass yield per plant (BYP), which is composed of straw yield per plant (SYP) and kernel yield per plant (KYP). HI is the ratio of KYP to BYP and reflects the allocation of photosynthetic products between grain and vegetative organs. Improving HI is one of the important goals in wheat breeding programs.

To date, many QTLs related to yield traits have been identified on all 21 chromosomes of wheat (Liu et al., 2020). However, due to the large genome size, hexaploid nature and high percentage of repetitive regions of wheat, most QTLs were mapped by a low-density genetic linkage map with large confidence interval, and only several yield-related QTLs have been fine mapped and cloned. A high-density genetic map based on an individual biparental mapping population would be of great value for high-resolution mapping and map-based cloning of a major targeted QTL. With the development of new sequencing technologies, high-density single nucleotide polymorphism (SNP) arrays technology has become a superior approach to construct genetic map and identify QTLs for yield-related traits in wheat. The high-density SNP assays including 9K (Wu et al., 2015), 35K (Allen et al., 2017), 55K (Ren et al., 2018), 90K (Wang et al., 2014), 660K (Cui et al., 2017) and 820K (Winfield et al., 2016), have become the best alternative to identify QTLs in wheat. For example, Cui et al. (2017) reported a high-density genetic map based on the 660K SNP array that was in good accordance with the released wheat genome assembly, providing a major resource for future genetic and genomic research. Using the high-density genetic map, a major QTL for KNS and eight putative additive QTL for PH were characterized (Cui et al., 2017; Zhang et al., 2017a). In addition, a high-density SNP genetic map is helpful to identify QTLs with major and stable effects. Converting the tightly linked SNP markers of QTLs into kompetitive allele specific PCR (KASP) markers that can be used for further validation in different genetic backgrounds, is important for marker-assisted transfer of these QTLs into elite breeding lines successfully. For instance, Liu et al. (2020) identified a QTL for kernel-related traits using a high-density genetic map based on the 660K SNP array and a KASP marker was developed for the QTL and verified by a natural population consisted of 141 cultivar/lines.

In our previous study, Xu et al. (2014) detected QTLs in a recombinant inbred line (RIL) population derived from ‘Xiaoyan 54’ and ‘Jing 411’ using a genetic map with 555 PCR-based markers and most QTLs were mapped in large confidence interval. Here, we used the Wheat 660K SNP array to re-genotype the ‘Xiaoyan 54 × Jing 411’RIL population and identify QTLs for 14 yield-related traits across six environments. Our objectives were to: (i) construct a high-density genetic linkage map; (ii) identify key QTLs that were significantly associated with yield-related traits in at least five environments; (iii) develop KASP marker based on the key QTLs and validate the loci in a diversity panel; (iv) predict candidate genes for the key QTLs. The results may contribute key QTLs and user-friendly markers for marker-assisted selection, which can facilitate yield improvement in wheat breeding and provide further insights into the genetic basis of yield-related traits in wheat.

## Materials and methods

### Plant materials

One hundred and eighty-two F_11_ RILs derived from a cross between wheat cultivars ‘Xiaoyan 54’ and ‘Jing 411’ were used for QTL mapping. ‘Xiaoyan 54’ was derived from the wheat founder parent ‘Xiaoyan 6’, which was released in 1980 and has been widely cultivated in the main wheat growing regions of China in the past 30 years (Li et al., 2008). ‘Jing 411’ was a widely grown cultivar in the Northern Winter Wheat Region of China in the 1990s (Zhuang, 2003).

A diversity panel composed of 190 wheat genotypes, including 42 wheat founder parents and widely grown cultivars at different decades since 1950, 32 elite cultivars widely grown in Huang-huai wheat region in recent years, 68 Xiaoyan 6-derivatived or related cultivars, and 48 accessions from Chinese wheat mini-core collections, was used to validate key loci detected in this study.

### Field trials and phenotyping

The trials were conducted at Luancheng Agroecosystem Experimental Station, the Chinese Academy of Sciences (37°53′15″N, 114°40′47″E, and elevation 50 m, located at the piedmont of the Taihang Mountains in the North China Plain).

The Xiaoyan 54/Jing 411 RIL population was planted during the 2006-2007 and 2007-2008 growing seasons. Three treatments were applied: low N (LN), low P (LP) and normal fertilized control (CK). Hereafter, ‘6LN’, ‘6LP’, ‘6CK’, ‘7LN’, ‘7LP’ and ‘7CK’ represent the six year × treatment trials, respectively. A randomized complete-block design was employed, with three separate adjacent blocks as the main plots for the three treatments and subplots for the 182 RILs and their parents (Xu et al., 2014). For each Xiaoyan 54/Jing 411 RIL, ten plants in the middle of the two internal rows in each plot were sampled for phenotyping. PH, BYP, KYP and SNPP were determined from the mean of the ten plants; SL, KNS, SSS, FSS, TSS and KWS were determined from the mean of the main spikes of the ten plants. TKW was evaluated after harvest by weighing 500 kernels in triplicate. HI was calculated as KYP/BYP, spikelet compactness (SCN) as TSS/SL and SYP as BYP-KYP.

The diversity panel was grown in four wheat growing seasons from 2012-2013 to 2015-2016. A split-plot design was employed, with three separate adjacent blocks as the main plots for the three replications, and subplots for the genotypes. Each accession was planted in four 150 cm-long rows, 25 cm apart, with 30 seeds per row. Seeds were hand planted at the beginning of October, and plants were harvested in the middle of next June at physiological maturity. For each accession of the diversity panel, nine yield-related traits including PH, SNPP, SL, KNS, TSS, FSS, SSS, TKW and KYP were evaluated.

### Map construction and QTL mapping

The 182 RILs as well as their parental cultivars were genotyped using the Wheat 660K SNP array (Sun et al., 2020). SNP flanking sequences were used to blast against the IWGSC wheat genome sequence (IWGSC RefSeq v1.1) to determine the physical locations of SNPs. SNP markers that had Call Rate > 97%, Heterozygote Rate < 20%, and Miner Allele Frequency > 5% were selected for map construction. To reduce the complexity of calculation, redundant markers (co-segregating markers) in the Xiaoyan 54/Jing 411 RIL population were removed using the BIN function in IciMapping 4.1 (http://www.isbreeding.net/) according to Meng et al. (2015). The unique SNP markers were sorted into linkage groups using the MAP function in IciMapping 4.1. The Kosambi mapping function was used to calculate genetic distances in centiMorgans (cM) with a LOD score of 3.5 and a recombination fraction of 0.3. Markers with no linkage or linkage groups with less than five markers were discarded in the subsequent analysis. The 21 chromosomes and the marker order were confirmed according to physical position of most SNPs in the Chinese Spring reference genome sequence of wheat (RefSeq v1.1) (Appels et al., 2018). MapChart 2.2 (http://www.biometris.nl/uk/Software/MapChart/) was used to draw the genetic map. QTL mapping was conducted using the MAP function in IciMapping 4.1 with the inclusive composite interval mapping of additive (ICIM-ADD) QTL method, a walk speed of 1.0 cM, and a stepwise regression probability of 0.001. The LOD threshold 3.0 was set to declare a significant QTL.

### Conversion of SNPs of key QTLs to KASP markers

Based on the flanking marker sequence of key QTL for yield-related traits that can be detected in at least five environments, eight SNPs were converted to KASP primers, which are specific for SNP genotyping (LGC Genomics LLC, Beverly, MA, USA). Newly designed KASP markers were evaluated for polymorphisms in reactions containing 5.0 µl water, 5.0 µl 2 × KASPar reaction mix, 0.14 µl assay mix, and 50 ng dried DNA, with a PCR profile of 94°C for 15 min (activation), followed by 10 cycles of 94°C for 20 s, 61-55°C for 60 s (drop 0.6°C per cycle), then 26 cycles of 94°C for 20 s and 55°C for 60 s. Fluorescence was measured as an end point reading at 37°C. KASP was performed in a BIO-RAD CFX Real-Time PCR system, and fluorescence was detected using Bio-Rad CFX Manage 3.1 software.

## Results

### Phenotypic variation and correlation analysis

**Figure 1:**
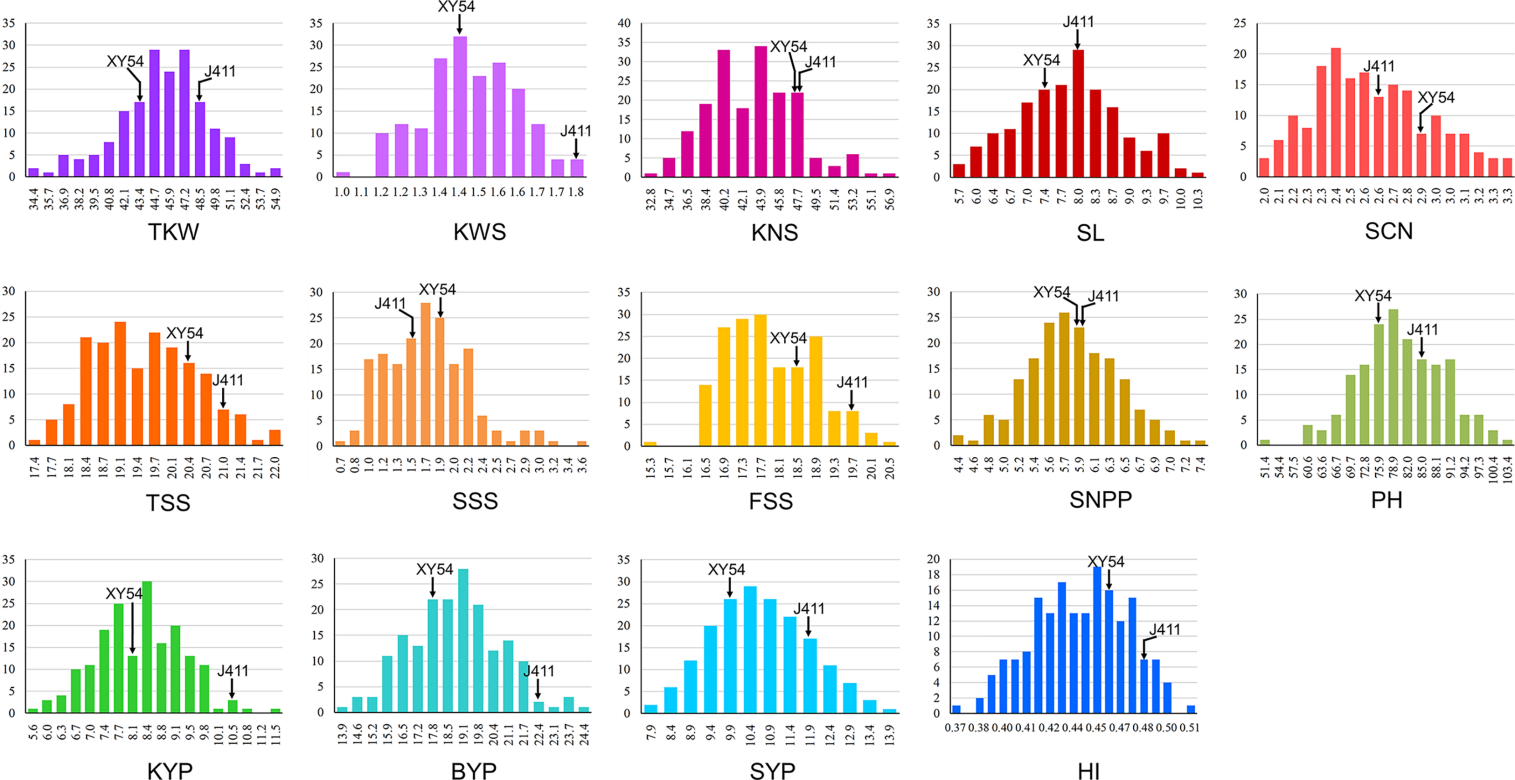
Histograms of the Xiaoyan 54/Jing 411 RIL population for thousand kernel weight (TKW), kernel weight per spike (KWS), kernel number per spike (KNS), spike length (SL), spikelet compactness (SCN), total spikelet number per spike (TSS), sterile spikelet number per spike (SSS), fertile spikelet number per spike (FSS), spike number per plant (SNPP), plant height (PH), kernel yield per plant (KYP), biomass yield per plant (BYP), straw yield per plant (SYP) and harvest index (HI).

**Figure 2:**
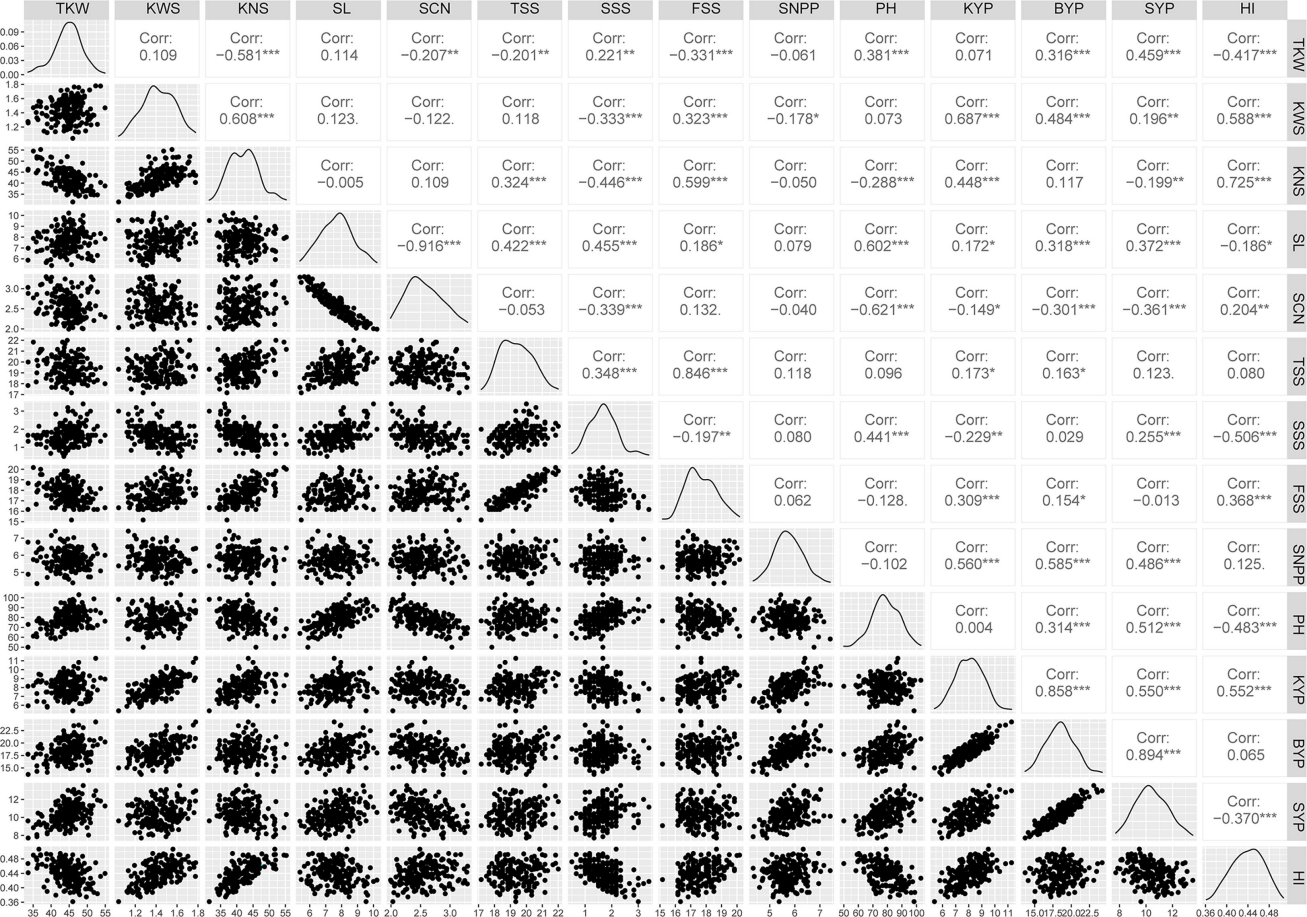
Correlation coefficients among yield-related traits of Xiaoyan 54/Jing 411 RIL population in six environments. Note: TKW, KWS, KNS, SL, SCN, TSS, SSS, FSS, SNPP, PH, KYP, BYP, SYP and HI are the same as **Table 2**; *, ** and *** indicate significant at 0.05, 0.01 and 0.001 levels, respectively.

Phenotypic performance of Xiaoyan 54/Jing 411 RIL population for the 14 yield-related traits is showed in **Figure 1** and **Supplementary Table S1**. Jing 411 had higher PH, SL, TKW, KWS, BYP, KYP and SYP across all the environments (**Figure 1**; **Supplementary Table S1**). Conversely, Xiaoyan 54 had higher spikelet compactness (SCN) and SNPP in four and three environments, respectively. In the RIL population, phenotypic values showed continuous variation and transgressive segregation (**Figure 1**), indicating polygenic inheritance. Estimated correlation coefficients among the 14 traits are showed in **Figure 2**. For the three yield traits, TKW had a significant and negative correlation with KNS, HI, FSS, SCN and TSS, and a significant positive correlation with SYP, PH and BYP. KNS was positively correlated with HI, KWS, FSS, KYP and TSS, and was negatively correlated with SSS, PH and SYP. SNPP had a positive correlation with BYP, KYP and SYP, and had a negative correlation with KWS. For the spike-related traits, SL had a significant and negative correlation with SCN, and a significant and positive correlation with PH, SSS, TSS, SYP, BYP, FSS and KYP. SCN was positively correlated with HI, and was negatively correlated with PH, SYP, SSS, BYP, TKW and KYP. TSS had a significant and positive correlation with FSS and SSS. KWS was positively correlated with KYP, HI, BYP, FSS and SYP, and was negatively correlated with SSS. Significant correlations were observed among BYP, KYP, SYP and SNPP. BYP had the highest positive correlation with SYP (r = 0.894), followed by BYP versus KYP (r = 0.858), BYP versus SNPP (r = 0.585), KYP versus SNPP (r = 0.560), KYP versus SYP (r = 0.550), and SYP versus SNPP (r = 0.486). HI had a positive correlation with KYP, and a negative correlation with SYP.

### Linkage map construction

**Figure 3:**
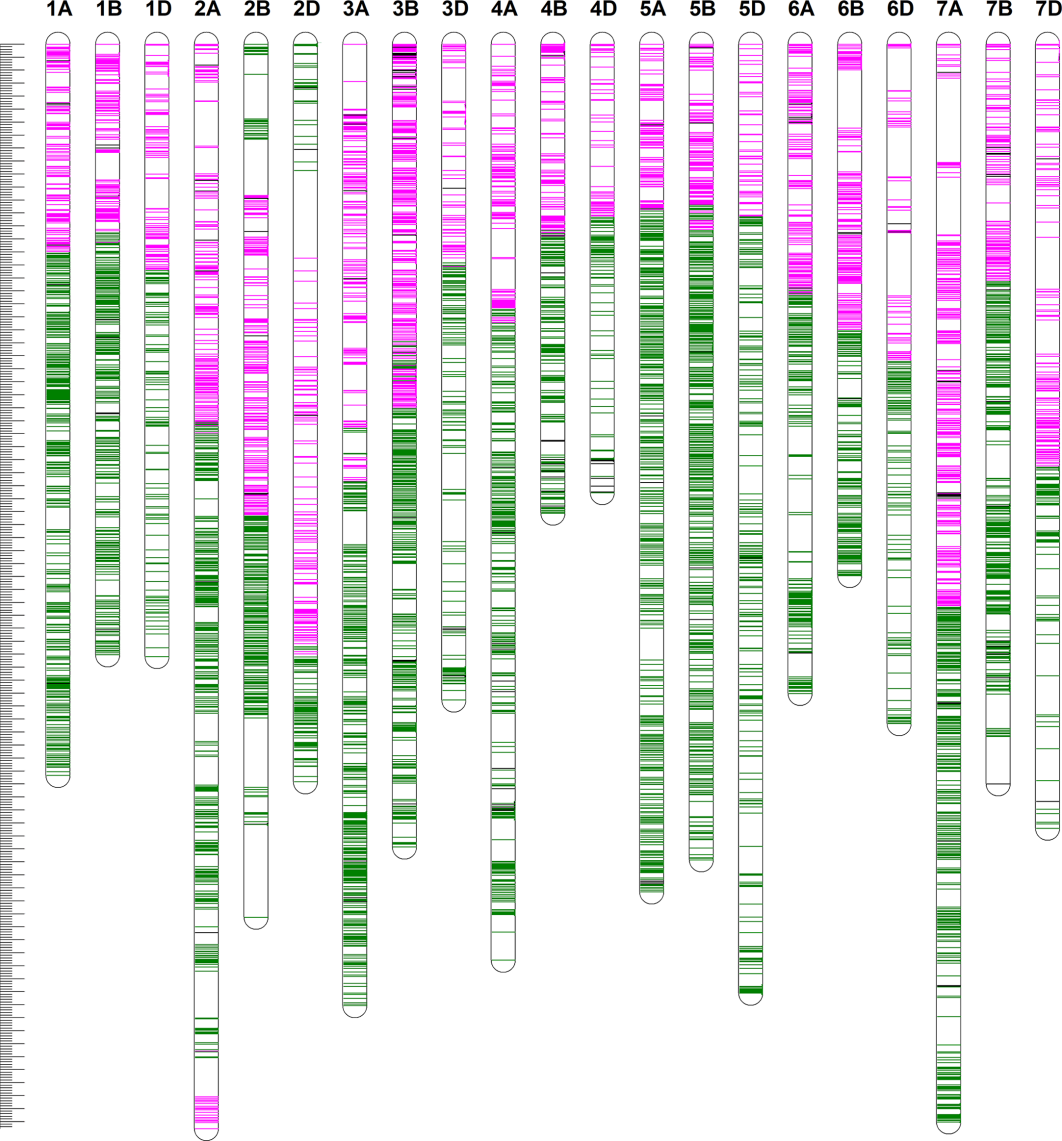
The high-density genetic linkage map of Xiaoyan 54/Jing 411 RIL population. For the redundant loci that showed co-segregation in the 182 RILs, only one unique informative marker is shown. The positions of the marker loci are indicated using a ruler on the left side. The names of the marker loci are listed to the right of the corresponding chromosomes. Loci in pink were best hits to Chinese Spring (CS) reference genome of the short arm of the corresponding chromosomes. Loci in green were best hits to CS reference genome of the long arm of the corresponding chromosomes. Loci in black were unknown.

**Figure 4:**
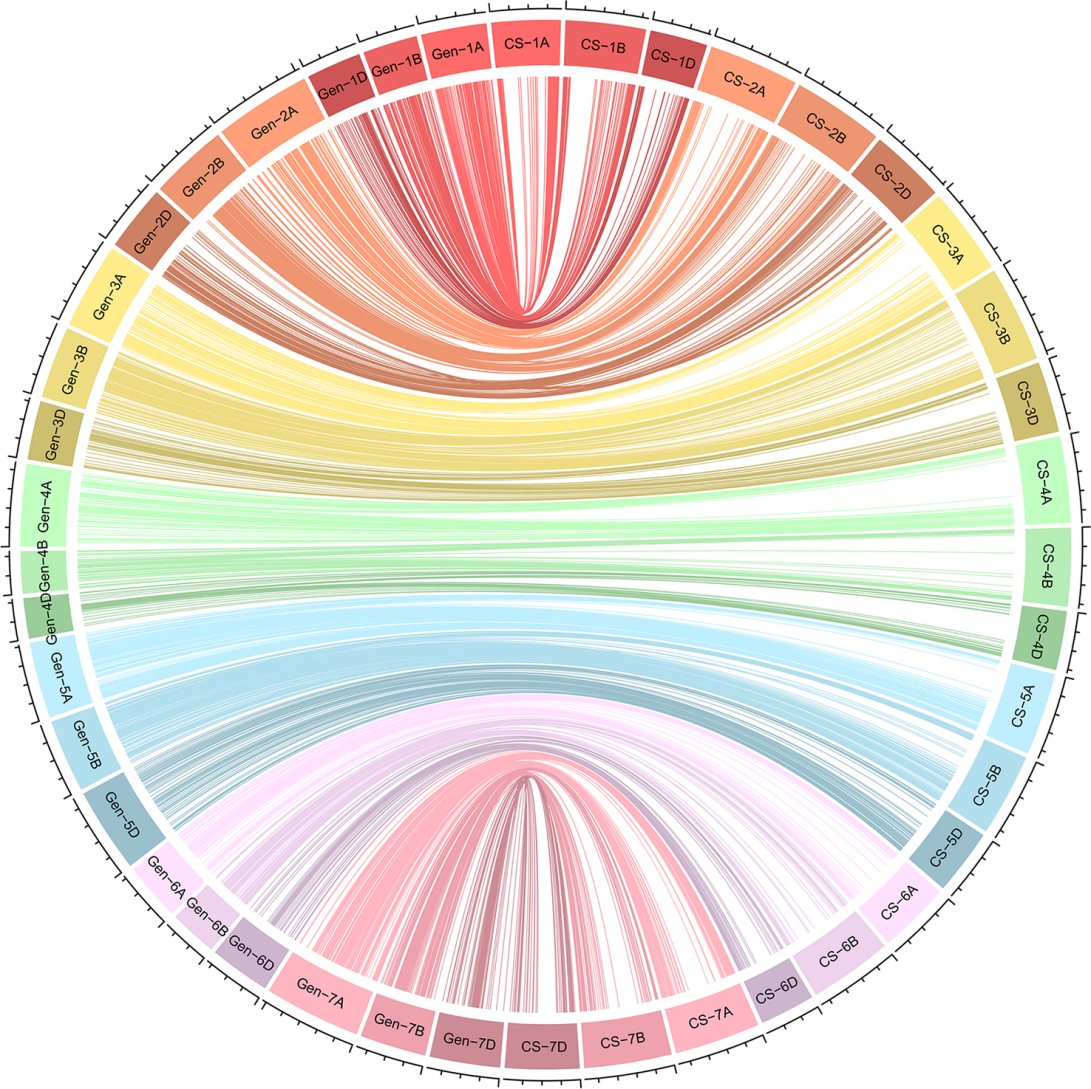
Schematic representation of the syntenic relationships between any one marker in wheat genetic and physical maps. Gen-1A to Gen-7D represent the 21 wheat chromosomal genetic maps released in this study; CS-1A to CS-7D represent the 21 wheat chromosomal physical maps of Chinese Spring reference genome. For the redundant loci that showed co-segregation in the 182 Xiaoyan 54/Jing 411 RILs, only one unique informative marker is shown.

We constructed a high-density linkage map with 7,542 unique loci spanning 6153.8 cM (**Figure 3**, **Table 1**). Of these loci, 6,987 were SNP markers derived from the Wheat 660K SNP array, and the remaining 555 markers were reported by Xu et al. (2014). The 7,542 markers distributed unevenly on the 21 chromosomes, and the number ranged from 132 for chromosome 4D to 565 for chromosome 3B. The genetic coverage of each chromosome varied from 172.71 cM (4D) to 417.71 cM (2A). Altogether, the markers mapping on the A genome (3,142) were more than those on the B genome (2,878), and much fewer markers (1,522) were mapped on the D genome. Seven gaps (>30 cM) were observed on chromosomes 2B, 2D and 7A (**Figure 3**; **Table 1**). Of these, the largest gap was found on 2B, which was 35.7 cM. The marker density of the individual chromosomes ranged from 0.55 cM/marker for 3B to 1.48 cM/marker for 6D with an average marker density of 0.82 cM/marker in the whole genetic map (**Table 1**). Markers mapped on the A and B genomes had a marker density of 0.77 and 0.65 cM/marker, while those mapped on the D genome had a density of 1.23 cM/marker. Based on the SNP flanking sequences, 6,987 markers were assigned to the wheat genome assembly (IWGSC RefSeq v1.1). SNP order in the present genetic map was in good agreement with that in the reference genome, except for chromosomes 2AL, 2BS and2DS, in which a segment inversion was identified (**Figures 3**, **4**).

**Table 1:** Summary information of the Xiaoyan 54/Jing 411 RIL high-density genetic map.

Chromosomes	Locus number	Map length (cM)	Average distance (cM)	Max distance (cM)
1A	460	281.69	0.61	8.52
1B	398	235.21	0.59	10.48
1D	215	235.86	1.10	11.50
2A	485	417.71	0.86	18.19
2B	420	336.30	0.80	35.66
2D	234	284.15	1.21	33.83
3A	457	370.32	0.81	14.31
3B	565	309.22	0.55	10.07
3D	219	252.58	1.15	15.96
4A	381	352.85	0.93	11.78
4B	234	180.62	0.77	7.01
4D	132	172.71	1.31	8.86
5A	453	326.46	0.72	12.22
5B	537	314.21	0.59	9.32
5D	285	365.45	1.28	12.86
6A	345	250.10	0.72	14.20
6B	314	204.58	0.65	22.42
6D	177	261.74	1.48	24.34
7A	561	415.14	0.74	32.39
7B	410	284.86	0.69	18.17
7D	260	302.01	1.16	19.93
Group1	1073	752.76	0.70	35.66
Group2	1139	1038.15	0.91	35.66
Group3	1241	932.12	0.75	15.96
Group4	747	706.17	0.95	11.78
Group5	1275	1006.13	0.79	12.86
Group6	836	716.42	0.86	24.34
Group7	1231	1002.01	0.81	32.39
GenomeA	3142	2414.27	0.77	32.39
GenomeB	2878	1865.00	0.65	35.66
GenomeD	1522	1874.50	1.23	33.83
Total	7542	6153.76	0.82	35.66

### QTL mapping analysis

A total of 285 significant QTLs were detected for the 14 examined traits in six environments, explaining 0.6-34.7% of the phenotypic variation (**Table 1**; **Supplementary Figure S1**, **Supplementary Table S2**). One hundred and twenty-nine QTLs showed positive effect with the Xiaoyan 54 allele. For the remaining 156 QTLs, the positive phenotype was derived from Jing 411. The QTLs that could be detected in three or more environments were regarded as environmentally stable QTLs. Twelve environmentally stable QTLs were identified in this study (**Table 2**). The QTLs detected for each trait were showed in **Supplementary Figure S1**.

**Table 2:** Environmentally stable QTLs for each yield-related trait of Xiaoyan 54/Jing 411 RIL population in six environments.

Traits	QTL	Evn.	Chr.	Site (cM)	LeftMarker	RightMarker	LOD	PVE^a^ (%)	Add^b^
Thousand kernel weight	*QTkw-1B.2*	6CK	1B	136	AX-109873144	AX-108946001	3.9	1.6	-0.67
(TKW)		6LN	1B	136	AX-109873144	AX-108946001	4.6	6.9	-1.01
		7CK	1B	136	AX-109873144	AX-108946001	6.0	4.5	-1.10
		7LN	1B	136	AX-109873144	AX-108946001	10.9	16.3	-1.69
		7LP	1B	136	AX-109873144	AX-108946001	7.1	14.4	-1.52
	*QTkw-4A.2*	6CK	4A	38	AX-94419996	AX-108742845	25.8	14.7	-2.01
		6LN	4A	38	AX-94419996	AX-108742845	7.4	11.7	-1.30
		7LP	4A	38	AX-94419996	AX-108742845	3.0	5.8	-0.95
	*QTkw-4D.1*	6LP	4D	36	AX-108735064	AX-110003964	4.1	3.5	1.00
		7LP	4D	36	AX-108735064	AX-110003964	4.6	9.4	1.22
		7CK	4D	38	AX-110003964	AX-109343336	4.5	3.5	0.96
		7LN	4D	39	AX-109343336	AX-111048443	3.5	4.7	0.90
Spike length	*QSl-2D.2*	6CK	2D	145	AX-111021196	AX-111561744	16.7	23.9	-0.52
(SL)		6LN	2D	145	AX-111021196	AX-111561744	21.4	34.7	-0.51
		6LP	2D	145	AX-111021196	AX-111561744	17.6	25.7	-0.52
		7CK	2D	145	AX-111021196	AX-111561744	26.9	28.2	-0.68
		7LN	2D	145	AX-111021196	AX-111561744	14.7	28.0	-0.51
		7LP	2D	145	AX-111021196	AX-111561744	15.9	28.3	-0.68
	*QSl-5A.1*	6CK	5A	107	AX-108742477	AX-108739527	7.5	9.5	-0.33
		6LN	5A	107	AX-108742477	AX-108739527	8.2	11.3	-0.29
		6LP	5A	107	AX-108742477	AX-108739527	8.8	11.5	-0.34
		7LN	5A	107	AX-108742477	AX-108739527	5.5	9.4	-0.29
Spikelet compactness	*QScn-2D.1*	6CK	2D	145	AX-111021196	AX-111561744	10.4	19.4	0.16
(SCN)		6LN	2D	145	AX-111021196	AX-111561744	10.1	14.2	0.12
		6LP	2D	145	AX-111021196	AX-111561744	11.1	22.9	0.16
		7CK	2D	145	AX-111021196	AX-111561744	7.7	15.0	0.11
		7LN	2D	145	AX-111021196	AX-111561744	14.1	14.5	0.15
		7LP	2D	145	AX-111021196	AX-111561744	7.9	13.6	0.12
	*QScn-5A.2*	6LN	5A	107	AX-108742477	AX-108739527	8.6	12.1	0.11
		6LP	5A	107	AX-108742477	AX-108739527	6.2	12.1	0.11
		7CK	5A	107	AX-108742477	AX-108739527	4.1	7.7	0.07
Total spikelet number per spike	*QTss-7A.2*	6CK	7A	300	AX-108794446	AX-108848493	4.7	11.2	-0.47
(TSS)		6LN	7A	300	AX-108794446	AX-108848493	12.3	22.4	-0.72
		6LP	7A	300	AX-108794446	AX-108848493	9.1	17.4	-0.52
		7CK	7A	300	AX-108794446	AX-108848493	8.8	17.8	-0.60
		7LN	7A	300	AX-108794446	AX-108848493	12.8	23.1	-0.74
Sterile spikelet number per spike	*QSss-2D.2*	6CK	2D	144	Xwmc112	AX-111021196	4.1	9.4	-0.22
(SSS)		6LP	2D	144	Xwmc112	AX-111021196	7.4	13.2	-0.31
		7LN	2D	145	AX-111021196	AX-111561744	4.6	9.3	-0.31
Plant height	*QPh-2D.1*	6CK	2D	145	AX-111021196	AX-111561744	11.4	12.5	-3.79
(PH)		6LN	2D	145	AX-111021196	AX-111561744	12.8	16.2	-4.14
		7CK	2D	145	AX-111021196	AX-111561744	16.8	21.0	-4.55
		7LN	2D	145	AX-111021196	AX-111561744	11.5	15.2	-3.49
		6LP	2D	146	AX-111561744	AX-111500777	15.2	14.9	-3.86
		7LP	2D	146	AX-111561744	AX-111500777	9.8	14.8	-3.54
	*QPh-4B.1*	6CK	4B	69	AX-109850058	AX-110713957	24.6	32.1	-6.01
		6LN	4B	69	AX-109850058	AX-110713957	23.0	33.4	-5.88
		6LP	4B	69	AX-109850058	AX-110713957	27.9	33.5	-5.74
		7CK	4B	69	AX-109850058	AX-110713957	20.6	27.6	-5.17
		7LN	4B	69	AX-109850058	AX-110713957	20.6	30.7	-4.90
		7LP	4B	69	AX-109850058	AX-110713957	13.1	20.5	-4.15
Harvest index	*QHi-5A.1*	6CK	5A	174	AX-108926070	AX-108801270	6.9	7.1	-0.01
(HI)		6LN	5A	174	AX-108926070	AX-108801270	7.5	12.1	-0.01
		6LP	5A	174	AX-108926070	AX-108801270	11.0	19.8	-0.02

a PVE indicates phenotypic variation explained by each QTL.

b The positive and negative additive values indicate Xiaoyan 54 and Jing 411 contributed increasing alleles for corresponding QTLs, respectively.

#### Kernel-related traits

Sixty-three QTLs for kernel-related traits (TKW and KWS) were detected on all chromosomes except for 7A, explaining 1.2-26.8% of the phenotypic variation (**Table 2**; **Supplementary Figure S1**, **Supplementary Table S2**). Of these, 23 QTLs showed positive effect with the Xiaoyan 54 allele, and 40 QTLs showed positive effect with the Jing 411 allele. Three environmentally stable QTLs for TKW were identified on chromosomes 1B, 4A and 4D (**Table 2**). The QTL *QTkw-1B.2* and *QTkw-4A.2* were significant in five and three environments, explaining 1.6-16.3% and 5.8-14.7% of the phenotypic variation, respectively. Jing 411 contributed effect for increased TKW at these loci. The QTL *QTkw-4D.1* was identified in four environments, explaining 3.5-9.4% of the phenotypic variation. Xiaoyan 54 contributed effect for increased TKW at the locus.

#### Spike-related traits

One hundred and sixteen QTLs for spike-related traits (KNS, SL, SCN, TSS, SSS and FSS) were identified on all chromosomes, explaining 2.1-34.7% of the phenotypic variation (**Table 2**; **Supplementary Figure S1**, **Supplementary Table S2**). Of these, 56 QTLs showed positive effect with the Xiaoyan 54 allele and 60 QTLs showed positive effect with the Jing 411 allele. Six environmentally stable QTLs were detected on chromosomes 2D (3), 5A (2) and 7A. Of these, *QSl-2D.2* was significant for SL across all the six environments, explaining 23.9-34.7% of the phenotypic variation. The QTL was also significant for SCN across all the eleven environments (*QScn-2D.1*), explaining 13.6-22.9% of the phenotypic variation. *QSl-5A.1* was significant for SL in four environments, explaining 9.4-11.5% of the phenotypic variation. The QTL was also significant for SCN in three environments (*QScn-5A.2*), explaining 7.7-12.1% of the phenotypic variation. Jing 411 contributed effect for increased SL and SCN at the two loci. *QTss-7A.2* was significant for TSS in five environments, explaining 11.2-23.1% of the phenotypic variation. Jing 411 contributed effect for an increased TSS at the locus. *QSss-2D.2* was significant for SSS in three environments, explaining 9.3-13.2% of the phenotypic variation. Jing 411 contributed effect for an increased SSS at the locus.

#### Plant architecture-related traits

Thirty-nine QTLs for plant architecture-related traits (SNPP and PH) were detected on all chromosomes except for 1B, 6A and 7D, explaining 0.6-33.5% of the phenotypic variation (**Table 2**; **Supplementary Figure S1**, **Supplementary Table S2**). Of these, 23 QTLs showed positive effect with the Xiaoyan 54 allele and 16 QTLs showed positive effect with the Jing 411 allele. Two environmentally stable QTLs *QPh-2D.1* and *QPh-4B.1* were both significant for PH across all the six environments, explaining 12.5-21.0% and 20.5-33.5% of the phenotypic variation, respectively. Jing 411 contributed effect for increased PH at these two loci.

#### Yield-related traits

Sixty-seven QTLs for yield-related traits (KYP, BYP, SYP and HI) were detected on all chromosomes except for 1A, 4D, 5B, 5D and 7D, explaining 3.8-19.8% of the phenotypic variation (**Table 2**; **Supplementary Figure S1**, **Supplementary Table S2**). Of these, 28 QTLs showed positive effect with the Xiaoyan 54 allele and 39 QTLs showed positive effect with the Jing 411 allele. The environmentally stable QTL *QHi-5A.1* was significant for HI in three environments, explaining 7.1-19.8% of the phenotypic variation. Jing 411 contributed effect for an increased HI at the locus.

### Development of KASP markers to validate the key loci

**Figure 5:**
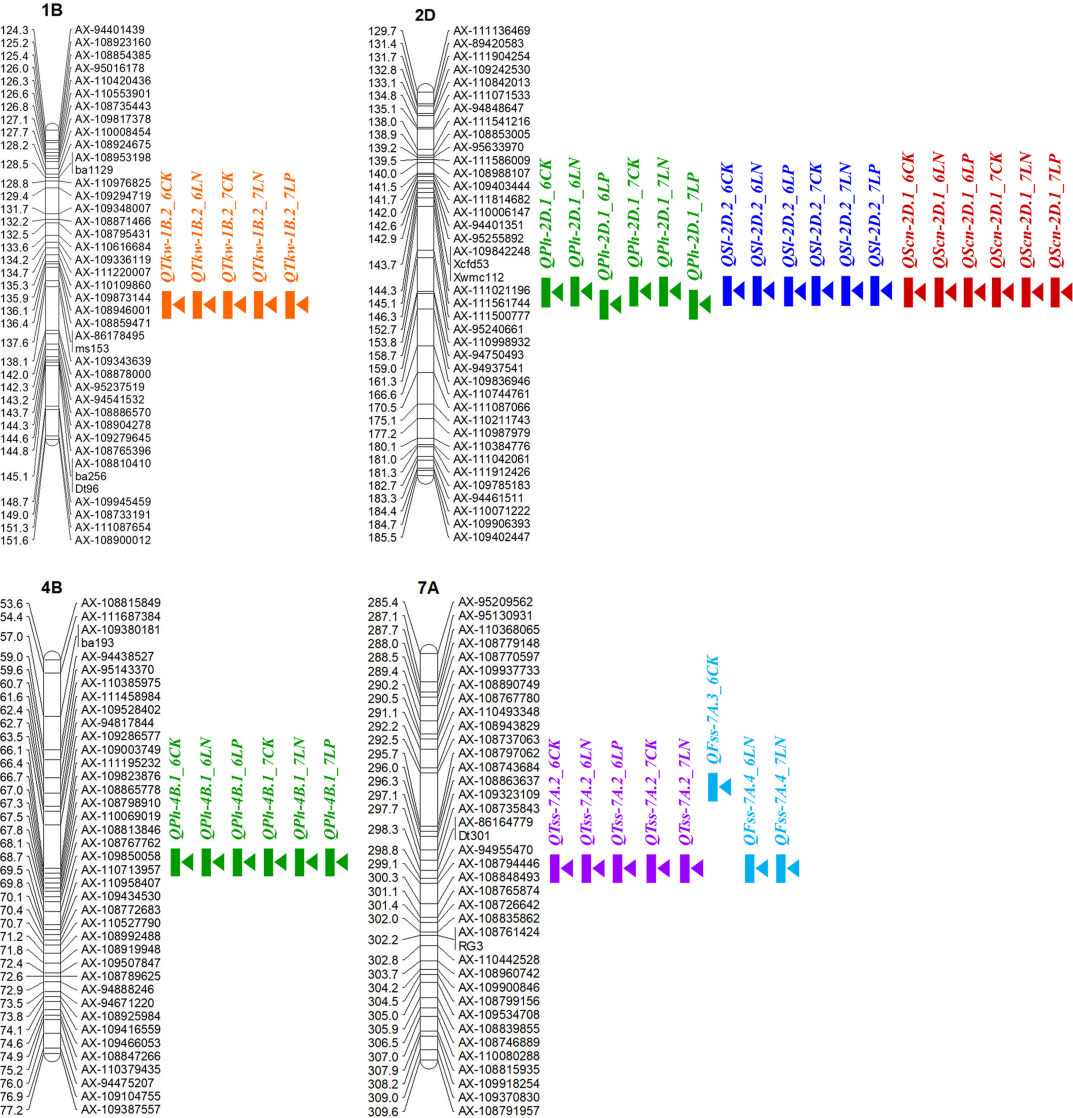
Locations of the four environmentally stable QTLs for yield-related traits in at least five environments. The short arms of the chromosomes are located at the top. The names of the marker loci and the QTL are listed on the right side of the corresponding chromosomes. The positions of the marker loci are listed on the left side of the corresponding chromosomes.

In this study, four environmentally stable QTLs *QTkw-1B.2*, *QPh-2D.1* (*QSl-2D.2*/*QScn-2D.1*), *QPh-4B.1* and *QTss-7A.2* were detected in at least five environments (**Figure 5**). Based on the flanking marker sequence of these QTLs, eight KASP markers were designed and tested for polymorphism in the diversity panel (**Supplementary Table S3**, **Supplementary Figure S2**). Two-tailed t-test was conducted for each marker in the diversity panel for yield-related traits. Apart from the QTL *QTkw-1B.2*, the other three QTLs were validated successfully in the diversity panel.

**Figure 6:**
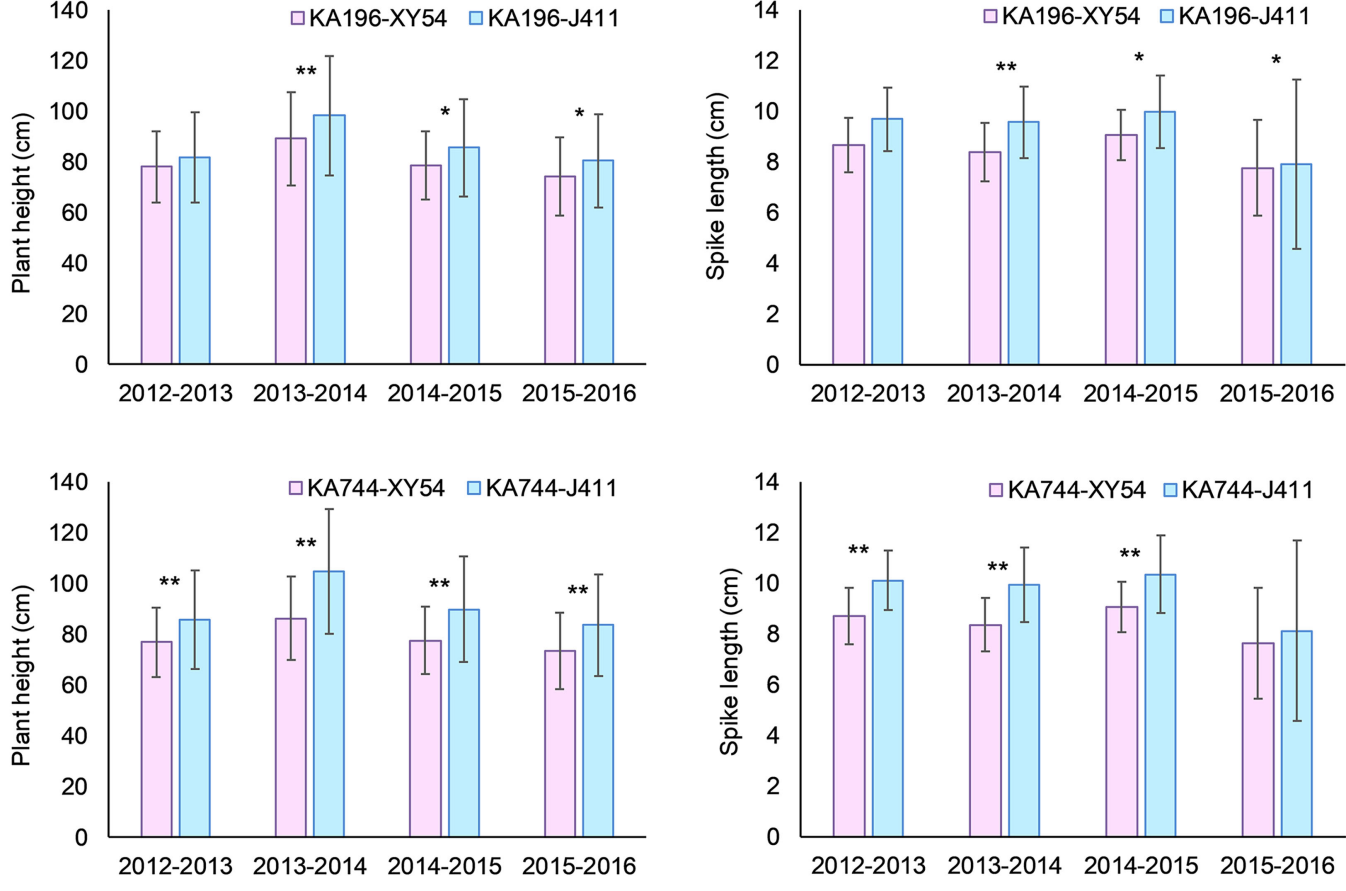
Mean difference in plant height (PH) and spike length (SL) between Xiaoyan 54 and Jing 411-derived alleles of KA196 and KA744 in the diversity panel. XY54 indicates the “Xiaoyan 54” allele; J411 indicates the “Jing 411” allele; * and ** indicate significant at 0.05 and 0.01 levels, respectively.

For the QTL *QPh-2D.1* (*QSl-2D.2*/*QScn-2D.1*) that was significant for PH, SL and SCN across all the six environments, two KASP markers KA196 and KA744 were developed based on the flanking SNP markers *AX-111021196* and *AX-111561744*, respectively (**Figure 5**; **Supplementary Table S3**, **Supplementary Figure S2**). In the diversity panel, KA196 and KA744 were significantly related to PH and SL in three to four environments (**Figure 6**). The accessions with Xiaoyan 54-derived alleles had lower PH and shorter SL, compared with the accessions with Jing 411-derived alleles. For the QTL *QPh-4B.1* that was significant for PH across all the six environments, two KASP markers KA058 and KA957 were developed based on the flanking SNP markers *AX-109850058* and *AX-110713957*, respectively (**Supplementary Table S3**, **Supplementary Figure S2**). In the diversity panel, the two KASP markers were significantly related to PH in four environments (**Supplementary Figure S3**). The accessions with Xiaoyan 54-derived alleles had lower PH, compared with the accessions with Jing 411-derived alleles. For the QTL *QTss-7A.3* that was significant for TSS in five environments, two KASP markers KA446 and KA493 were developed based on the flanking SNP markers *AX-108794446* and *AX-108848493*, respectively (**Supplementary Table S3**, **Supplementary Figure S2**). In the diversity panel, the KASP marker KA493 were significantly related to TSS (**Supplementary Figure S4**). The accessions with Xiaoyan 54-derived alleles had more TSS than those with Jing 411-derived alleles.

### Prediction of candidate genes in the four key loci

In the present study, four important loci were detected in at least five environments. Of these, the QTL *QTkw-1B.2* was identified for TKW in five environments (**Figure 5**). The confidence interval of *QTkw-1B.2* was bounded by SNP markers *AX-109873144* and *AX-108946001*, corresponding to a physical distance of ~ 1.3 Mb (627,922,061- 629,168,892 bp, RefSeq v1.1), which contained 23 high-confidence annotated genes (**Supplementary Table S4**). Among these genes, *TraesCS1B01G396600*, which encodes the U4/U6 small nuclear ribonucleoprotein Prp31, showed higher expression level in developing wheat grain (Ramirez-Gonzalez et al., 2018) and may be the candidate gene for *QTkw-1B.2*. Further research is needed to firmly conclude the candidate gene. The QTL *QPh-2D.1* (*QSl-2D.2/QScn-2D.1*) was significantly related to PH, SL and SCN across all the six environments (**Figure 5**). In the diversity panel, two KASP markers based on the flanking SNP markers of the QTL were significantly related to PH and SL (**Figure 6**). The confidence interval of *QPh-2D.1* (*QSl-2D.2/QScn-2D.1*) was bounded by SNP markers *AX-111021196* and *AX-111561744*, corresponding to a physical distance of ~ 0.9 Mb (22,498,824- 23,416,219 bp, RefSeq v1.1), which contained 45 high-confidence annotated genes (**Supplementary Table S5**). The famous reduced height (Rht) gene *Rht8* was reported in this interval (Chai et al., 2019). Chai et al. (2022) isolated the candidate gene *TraesCSU02G024900* for *Rht8 via* map-based gene cloning. The gene encodes a protein containing a zinc finger BED-type motif and an RNase H-like domain that regulates plant height *via* influencing bioactive gibberellin biosynthesis. Similar results were found by Xiong et al. (2022). Therefore, *TraesCSU02G024900* might be the candidate gene for *QPh-2D.1.* The QTL *QPh-4B.1* was significant for PH across all the six environments (**Figure 5**). In the diversity panel, two KASP markers were significantly related to PH in four environments (**Supplementary Figure S3**). The confidence interval of *QPh-4B.1* was bounded by SNP markers *AX-109850058* and *AX-110713957*, corresponding to a physical distance of ~ 2.0 Mb (40,904,736- 42,912,713 bp, RefSeq v1.1), which contained 18 high-confidence annotated genes (**Supplementary Table S6**). Among these genes, *TraesCS4B02G053600* is an ortholog of the rice gene *Decrease in DNA Methylation 1* (*OsDDM1*), which was reported to be related to dwarf phenotypes (Higo et al., 2012). The QTL *QTss-7A.3* was significant for TSS in five environments (**Figure 5**). In the diversity panel, the KASP marker KA493 were significantly related to TSS (**Supplementary Figure S4**). The confidence interval of *QTss-7A.3* was bounded by SNP markers *AX-108794446* and *AX-108848493*, corresponding to a physical distance of ~ 2.2 Mb (672,893,634- 675,112,612 bp, RefSeq v1.1), which contained 27 high-confidence annotated genes (**Supplementary Table S7**). Among these genes, *TraesCS7A02G481600*, which is the A-genome homeolog of *WHEAT ORTHOLOG OF APO1* (*WAPO-A1*), was reported to be the leading candidate gene for *QTss-7A.3* affecting spikelet number per spike (Kuzay et al., 2019, Kuzay et al., 2022).

## Discussion

### Comparison of the major QTLs with previous observations

In this study, we detect 285 QTLs for 14 yield-related traits using a high-density linkage map. Of these, the QTL *QTkw-1B.2* was identified for TKW in five environments and was located in the position interval 627.9-629.2 Mb of chromosome 1B (RefSeq v1.1). In the previous studies, Li et al. (2019) performed genome-wide association study (GWAS) in 166 wheat cultivars and identified a significant QTL associated with TKW in the interval 658.7-662.5 Mb of 1B based on the Wheat 90K and 660K SNP arrays. Quarrie et al. (2005) detected a QTL for TKW near the position 555.93 Mb using a doubled-haploid (DH) population derived from the cross of Chinese Spring and SQ1. Cui et al. (2014) also identified a QTL for TKW near the position 555.93 Mb using three related RIL populations. Zanke et al. (2015) performed GWAS in a wheat panel and detected three significant SNP loci on 1B associated with TKW. Pang et al. (2020) conducted a large-scale GWAS using a panel of 768 wheat cultivars and detected a significant QTL for TKW in the interval 667.9-668.1 Mb of 1B under three environments. We located the three SNP loci at the positions 14.1Mb, 560.5Mb and 649.1Mb of 1B by BLAST-searching against the Chinese Spring reference genome sequence. By comparison of the QTL position with previous observations, we found that the QTL *QTkw-1B.2* identified in this study was different from those from previous studies and may be a novel QTL for TKW, which represented a valuable target for map-based cloning and marker-assisted selection to enhance grain yield in wheat breeding.

The QTL *QPh-4B.1* was significant for PH across all the six environments. In the diversity panel, two KASP markers KA058 and KA957 were significantly related to PH in four environments. The QTL was located in the position interval 40.9-42.9 Mb of chromosome 4B (RefSeq v1.1). The “Green revolution” gene *Rht-B1b* was reported at 30.86 Mb of 4B (Xu et al., 2019), which is different from the QTL *QPh-4B.1*. The other height-reducing genes *Rht3* (*Rht-B1c*), *Rht11* (*Rht-B1e*) and *Rht17* (*Rht-B1p*) on 4B were allelic to *Rht-B1b* (Zhang et al., 2021). In the previous studies, Zhang et al. (2017a) detected a QTL for PH near the locus *Rht-B1b* in multi-environments using a 660K high-density map, and we located it to the 27.4-28.9 Mb by BLAST-searching against the Chinese Spring reference genome sequence. Li et al. (2018) identified a QTL for PH at 25.8 Mb of 4B in three bread wheat populations using the Wheat 90K SNP array. Tadesse et al. (2015) detected a locus associated with PH at the position 68.1 Mb of 4B using a total of 120 elite wheat accessions. Guo et al. (2015) identified a locus *Xgwm495* associated with PH at the position 482.8 Mb using a set of 230 wheat cultivars by association mapping. Zhuang et al. (2021) cloned a PH-related gene *TaSRL1* from wheat at the position 585.8 Mb of 4B. By comparison of the QTL *QPh-4B.1* position with previous observations, we found that the QTL for PH detected in this study was different from those from previous studies and may be a novel QTL, which deserved for further studies including positional cloning and marker-assisted selection.

In this study, QTL *QPh-2D.1* (*QSl-2D.2*/*QScn-2D.1*/*QSss-2D.2*/*QTss-2D.1*) was identified for PH, SL and SCN across all the six environments, for SSS in three environments and for TSS in one environment. Two KASP markers based on the flanking SNP markers of the QTL were significantly related to PH and SL in the diversity panel (**Figure 6**). The QTL was located in the position interval 22.5-23.4 Mb of chromosome 2D (RefSeq v1.1). In the previous studies, the famous Rht gene *Rht8* was reported to be located on the same genomic interval (Chai et al., 2019). Chai et al. (2022) isolated the candidate gene for *Rht8 via* map-based gene cloning and confirmed that loss of RNHL-D1 is responsible for semi-dwarf trait in *Rht8*-carrying wheat plants. Xiong et al. (2022) identified two new semi-dwarf wheat mutants that are allelic to *Rht8* and revealed the complexity and evolutionarily history of *Rht8* in common wheat. Zhai et al. (2016) detected a pleiotropic QTL for PH, SL and SSS at the position 23.0 Mb on 2D using the RIL population derived from Yumai 8679 and Jing 411. Xu et al. (2014) identified a QTL cluster in the interval 23.0-24.7 Mb of 2D controlling PH, SL, SSS and TSS using Xiaoyan 54/Jing 411 RIL population. Ma et al. (2007) identified a major QTL for SL on 2DS using Nanda 2419/Wangshuibai RIL population. Then the QTL was precisely mapped near the position 23.0 Mb (Wu et al., 2013). Zhou et al. (2017) detected a QTL for SL in the interval 22.9-23.7 Mb of 2D using a soft red winter wheat DH population. Using another DH population, Sourdille et al. (2003) detected a QTL for SL in the interval 20.4-24.3 Mb of 2D. Ma et al. (2018) identified a major QTL for FSS in the similar position through GWAS and found the QTL could affect SL, TSS and SSS. Therefore, it seems that the QTL on 2D in this study contained *Rht8* gene and was a pleiotropic locus that played an important role in affecting PH, SL, TSS, SSS and FSS.

The QTL *QTss-7A.3* (*QFss-7A.4*) was detected for TSS and FSS in five and two environments, respectively. In the diversity panel, the KASP marker KA493 were significantly related to TSS (**Supplementary Figure S4**). The QTL was located in the position interval 672.9- 675.1 Mb of chromosome 7A (RefSeq v1.1). In the previous studies, Xu et al. (2014) identified a QTL for TSS, FSS and SSS in the interval 668.0-679.9 Mb of 7A using Xiaoyan 54/Jing 411 RIL population. Zhang et al. (2018a) identified a SNP for TSS at the position 674.3 Mb of 7A in a spring wheat panel and validated the SNP in a biparental population. Boeven et al. (2016) conducted GWAS and detected a QTL for TSS near the position 674.3 Mb of 7A in a diverse set of 209 winter bread wheat lines. Faris et al. (2014) identified a QTL for TSS in the interval 671.4-674.3 Mb of 7A using a RIL population derived from a cross between a cultivated emmer accession and a durum wheat cultivar. Voss-Fels et al. (2019) performed GWAS in a panel of 220 winter wheats and detected a highly significant QTL for TSS in the interval 672.0-674.3 Mb of 7A. All these results suggested that the QTL on 7A was a key locus and showed significant effects for TSS in various environments. What’s more, Kuzay et al. (2019) delimited this QTL to an 87-kb region (674,019,191-674,106,327 bp, RefSeq v1.1) containing four candidate genes and identified *WAPO-A1* as the most promising candidate gene. Loss-of-function mutations in the *WAPO-A1* gene reduced TSS and additional transgenic copies of this gene increased TSS. Haplotype analysis showed that H2 variant is associated with the largest increases in TSS and KNS in field experiments (Kuzay et al., 2022). Therefore, the utilization of the *WAPO-A1* variant represents a promising opportunity to improve grain yield in wheat.

### The high-density linkage map and comparative mapping

Constructing a high-quality and saturated genetic map is the prerequisite of QTL mapping. Based on the new sequencing technologies, a great number of SNPs have been identified and used for genetic map construction in wheat. Some high-density maps were reported using the high-throughput microarray genotyping method, such as the Wheat 9K, 90K and 660K arrays (Wu et al., 2015; Zhai et al., 2016; Cui et al., 2017). Sun et al. (2020) evaluated seven widely used high-throughput wheat arrays (Wheat 9K, 15K, 35K, 55K, 90K, 820K and 660K arrays) in terms of their SNP number, distribution, density, associated genes, heterozygosity, and application. The results suggested that the Wheat 660K SNP array is reliable and cost-effective and may be the best choice for targeted genotyping and marker-assisted selection in wheat genetic improvement. In the present study, we genotyped the Xiaoyan 54/Jing 411 RIL population using the Wheat 660K SNP array and developed a high-density linkage map of 7,542 polymorphisms markers. Based on the SNP flanking sequences, we assigned the markers to the reference genome of Chinese Spring. As shown in **Figure 3**, the genetic and physical positions of the mapped markers were generally in agreement. The SNP order in the present genetic map was also in good agreement with that in the physical position (**Figure 4**), which prompted us to search for candidate genes of major targeted QTLs. Using the high-density genetic map of Xiaoyan 54/Jing 411 RIL population, we identified 285 significant QTLs for the 14 examined traits in six environments, explaining 0.6-34.7% of the phenotypic variation (**Supplementary Figure S1**; **Table 1**; **Supplementary Table S2**). The number of QTLs significantly related to each trait ranged from 11 to 41. Xu et al. (2014) genotyped the Xiaoyan 54/Jing 411 RIL population using gel-based markers and constructed a genetic linkage map with 555 polymorphic loci. Based on the genetic map, 89 QTLs for the same 14 yield-related traits were identified and the number of QTLs significantly related to each trait ranged from 2 to 14, which was much less than those detected in this study. The results showed that more QTLs could be identified using a high-density linkage map. What’s more, Xu et al. (2014) identified a major QTL (*QTss-7A*) for TSS in the interval *Xbarc192-Xbarc253* in five environments, which could explain 7.1-20.5% of the phenotypic variation. We located the two flanking markers at the positions 668.0 Mb and 680.0 Mb of 7A (RefSeq v1.1). In this study, we also detected a QTL for TSS in five environments, explaining 11.2-23.1% of the phenotypic variation. The QTL was located in the position interval 672.9- 675.1 Mb of 7A (RefSeq v1.1). By comparison of the positions the two QTLs, we found that the confidence intervals of QTLs identified using a high-density linkage map were much smaller than those identified using a low-density map. It is worth noting that Xu et al. (2014) detected a QTL for TKW on chromosome 1B in 7LN and 7LP two environments, explaining 9.4% and 9.9% of the phenotypic variation, respectively, while in this study, the QTL *QTkw-1B.2* was significant in five environments, explaining16.3% and 14.4% of the phenotypic variation in the 7LN and 7LP environments, respectively, and 1.6-6.9% of phenotypic variation in other three environments. These results showed that QTLs with lower phenotypic variance explained could be detected using a high-density linkage map. Taken together, it is more efficient to detect QTLs for yield-related traits using an improved high-density linkage genetic map.

### Potential implications in wheat breeding

How to increase wheat yield has been a major focus of most wheat breeders. Wheat yield is significantly influenced by environment, which presents a major challenge to select high-yielding lines at the early stages of breeding programs. In contrast, yield-related traits, such as TKW, SL, PH and TSS, are less influenced by environment. Consequently, more effort has been put into yield-related traits to improve wheat yield. Identification of stable major QTLs for yield-related traits is of high importance in molecular breeding. In the present study, we detected four environmentally stable QTLs for yield-related traits in at least five environments using a high-density genetic map based on the Wheat 660K SNP array. Of these, the QTL *QPh-2D.1* (*QSl-2D.2/QScn-2D.1*) was identified for PH, SL and SCN across all the six environments. The QTL *QTss-7A.3* was detected for TSS in five environments. By comparison of the QTLs with previous observations, we found that the two QTLs showed constant effects on their corresponding yield-related traits in different genetic backgrounds and were strongly selected in breeding. The QTLs *QTkw-1B.2* and *QPh-4B.1* were identified for TKW and PH in five and six environments, respectively. The two QTLs were different from those from previous studies and might be novel QTLs. In the diversity panel, *QPh-2D.1* (*QSl-2D.2/QScn-2D.1*), *QTss-7A.3* and *QPh-4B.1* were validated successfully by developing KASP markers. These major QTLs represented a valuable target for marker-assisted selection to improve yield-related traits. The availability of time-saving and cost-effective KASP markers could facilitate their use in wheat breeding. With the application of high-density linkage maps in QTL detection and user-friendly flanking markers, wheat breeding by molecular design is not a distant goal.

## Conclusion

We constructed a high-density genetic map using an RIL population with the Wheat 660K SNP array. The genetic map showed high collinearity with the wheat genome assembly. Using the high-density genetic map, we conducted QTL mapping for 14 yield-related traits in six environments. Four major QTLs, *QTkw-1B.2*, *QPh-2D.1* (*QSl-2D.2*/*QScn-2D.1*), *QPh-4B.1* and *QTss-7A.3*, were detected in at least five environments. Of these, *QPh-2D.1* (*QSl-2D.2*/*QScn-2D.1*), *QPh-4B.1* and *QTss-7A.3* were successfully validated in the natural population based on the developed KASP markers. By comparing with results from previous studies, we found that *QTkw-1B.2* and *QPh-4B.1* should be novel QTLs. The identified QTLs and the developed KASP marker will be valuable for further positional cloning and marker-assisted selection in wheat breeding programs.

## Data availability statement

The original contributions presented in the study are included in the article/**Supplementary Material**. Further inquiries can be directed to the corresponding authors.

## Author contributions

DA, DL, YT and AZ conceived the research. DL, YT and AZ constructed the RIL population. FM, YX and RW performed phenotypic assessments. FM and YX carried out statistics analysis, QTL mapping and developed the KASP markers. FM wrote the manuscript. DA and DL supervised and revised the manuscript. All authors read and approved the final manuscript.
