# Supplementary material for: Identification of major QTLs for yield-related traits with improved genetic map in wheat
Source: Front Plant Sci. 2023 Mar 17;14:1138696. doi: 10.3389/fpls.2023.1138696 (PMC10063875; doi:10.3389/fpls.2023.1138696)
Supplement: Supplementary file 8 [file DataSheet_1.pdf]

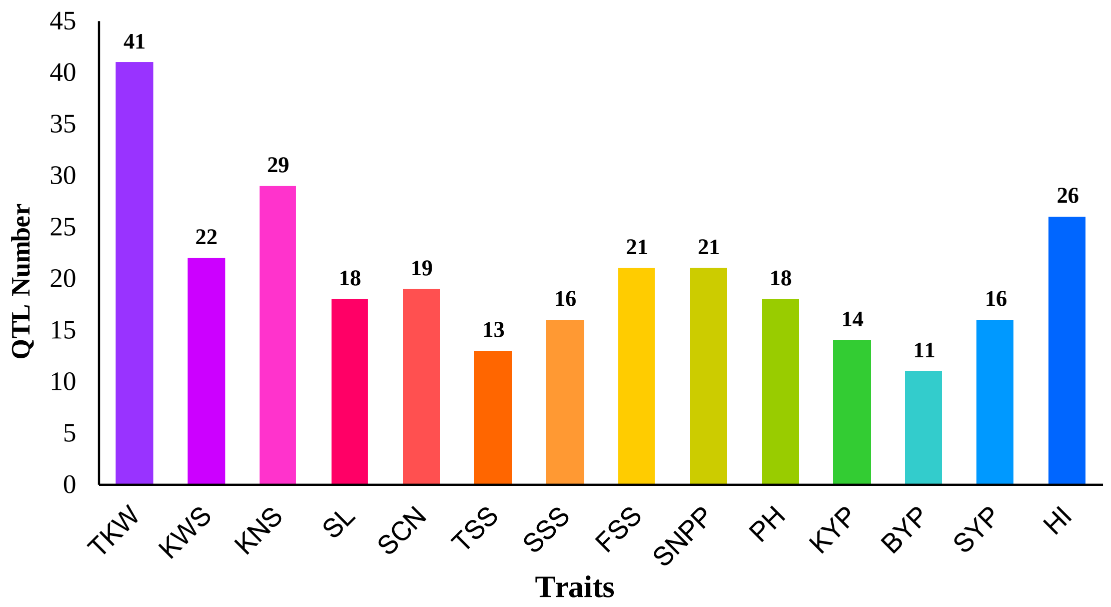

Fig. S1 The numbers of QTLs for 14 yield-related traits detected in this study. PH plant height, SL spike length, SCN spikelet compactness, SNPP spike number per plant, KNS kernel number per spike, TKW thousand kernel weight, KWS kernel weight per spike, TSS total spikelet number per spike, SSS sterile spikelet number per spike, FSS fertile spikelet number per spike, BYP biomass yield per plant, KYP kernel yield per plant, SYP straw yield per plant and HI harvest index

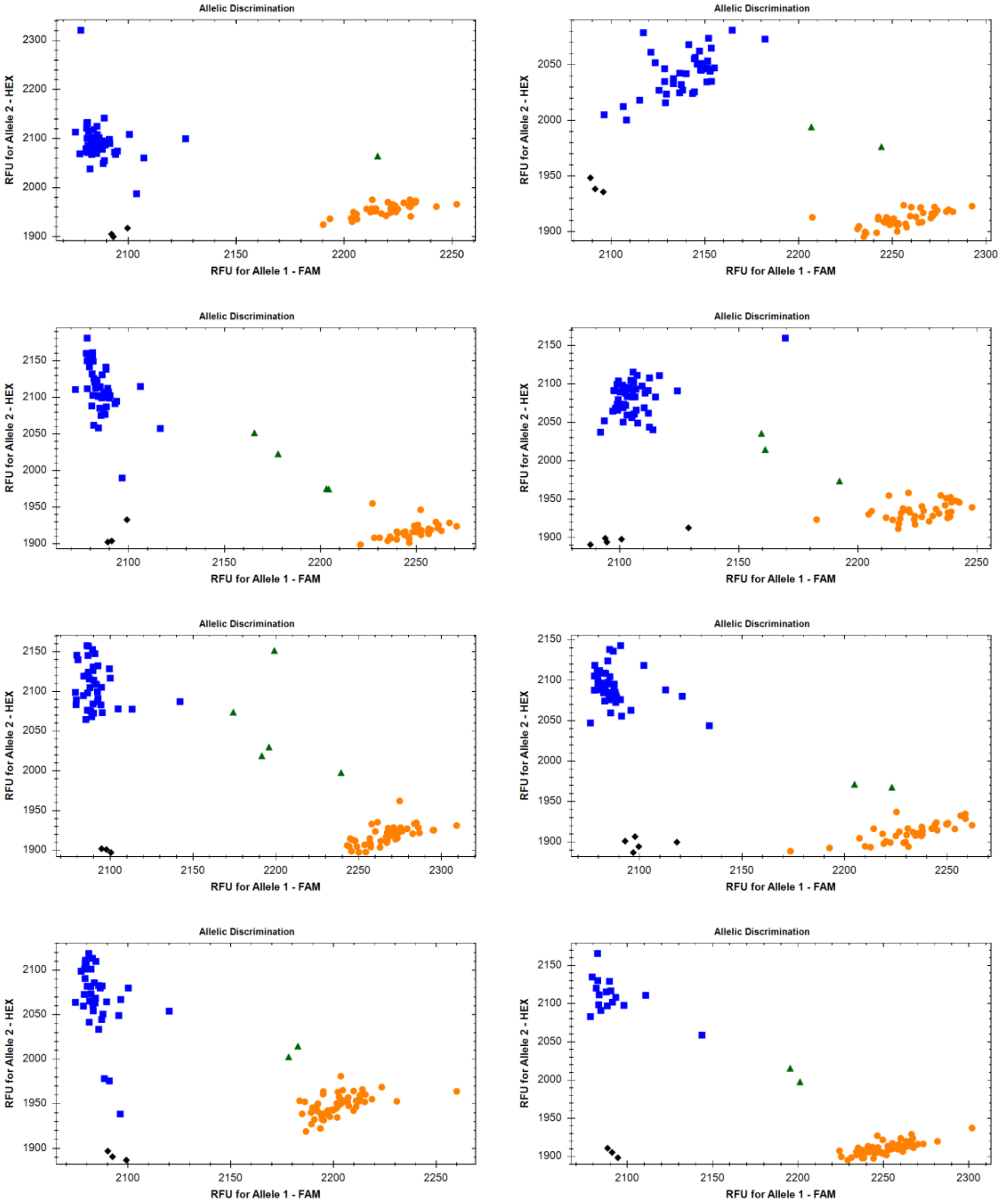

Fig. S2 Scatter plots for the eight KASP assays (KA144, KA001, KA196, KA744, KA058, KA957, KA446 and KA493）in diversity panel showing clustering of varieties on the X- (FAM) and Y- (HEX) axes. Varieties colored blue have the HEX-type allele, varieties colored red have the FAM-type allele, varieties colored green are heterozygote that have the two types of alleles, black dots represent the NTC (non-template control)

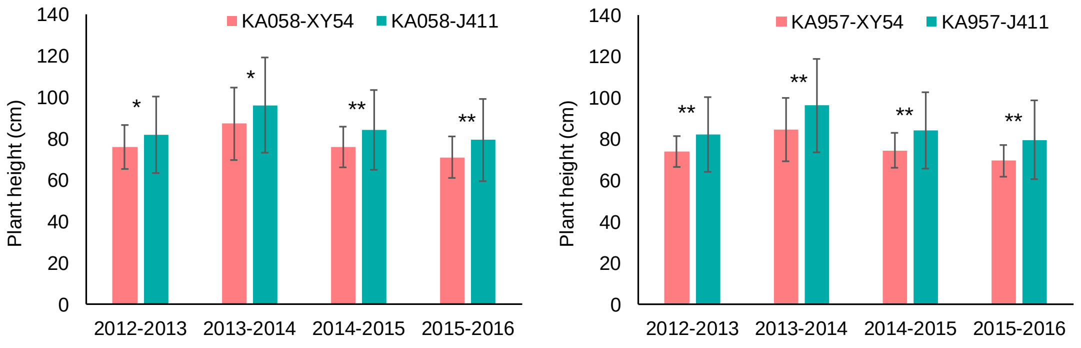

Fig. S3 Mean difference in plant height (PH) between Xiaoyan 54 and Jing 411-derived alleles of KA058 and KA957 in the diversity panel. XY54 indicates the "Xiaoyan 54" allele; J411 indicates the "Jing 411" allele; * and ** indicate significant at 0.05 and 0.01 levels, respectively

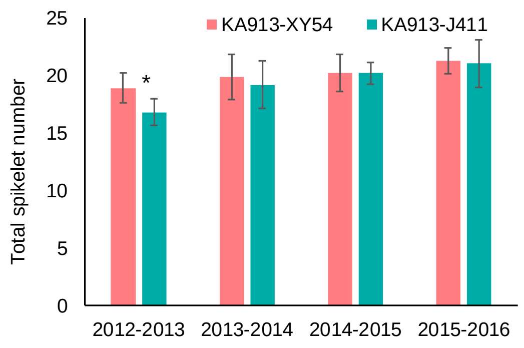

Fig. S4 Mean difference in total spikelet number per spike (TSS) between Xiaoyan 54 and Jing 411-derived alleles of KA913 in the diversity panel. XY54 indicates the "Xiaoyan 54" allele; J411 indicates the "Jing 411" allele; * indicates significant at 0.05 level
